# Supplementary material for: Anti-Quorum-Sensing Potential of Ethanolic Extracts of Aromatic Plants from the Flora of Cyprus
Source: Plants (Basel). 2022 Oct 7;11(19):2632. doi: 10.3390/plants11192632 (PMC9572961; doi:10.3390/plants11192632)
Supplement: Supplementary file 1 [file plants-11-02632-s001.zip › plants-1858476-supplementary.pdf]

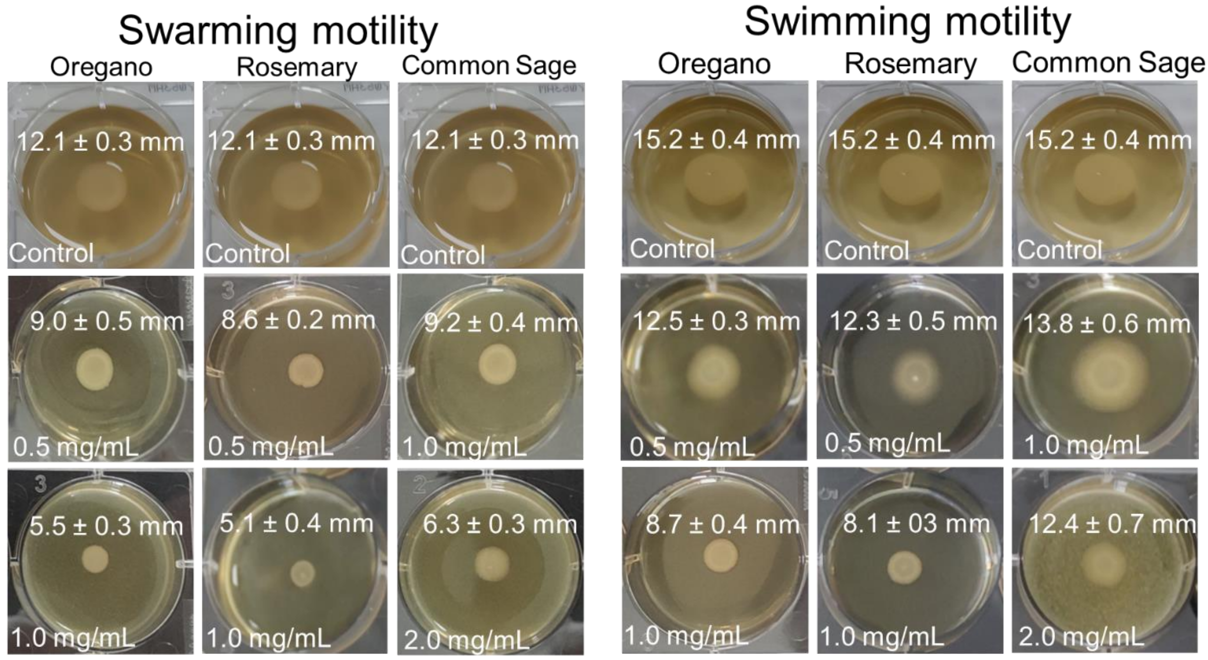

Figure S1: Inhibition of swarming and swimming motilities of *E. coli* MG1655 by different concentrations of the ethanolic extracts. *E. coli* MG1655 was grown on LB agar in the absence and in the presence of ethanolic extracts of oregano (at 0.5 mg/mL or 1 mg/mL), rosemary (at 0.5 mg/mL or 1 mg/mL) or common sage (at 1 mg/mL or 2 mg/mL) overnight at 37°C. The next morning the swimming and swarming migrations were recorded by measuring the diameter of swarm fronts or swim zones. Results are presented as mean ± SD, n = 3.

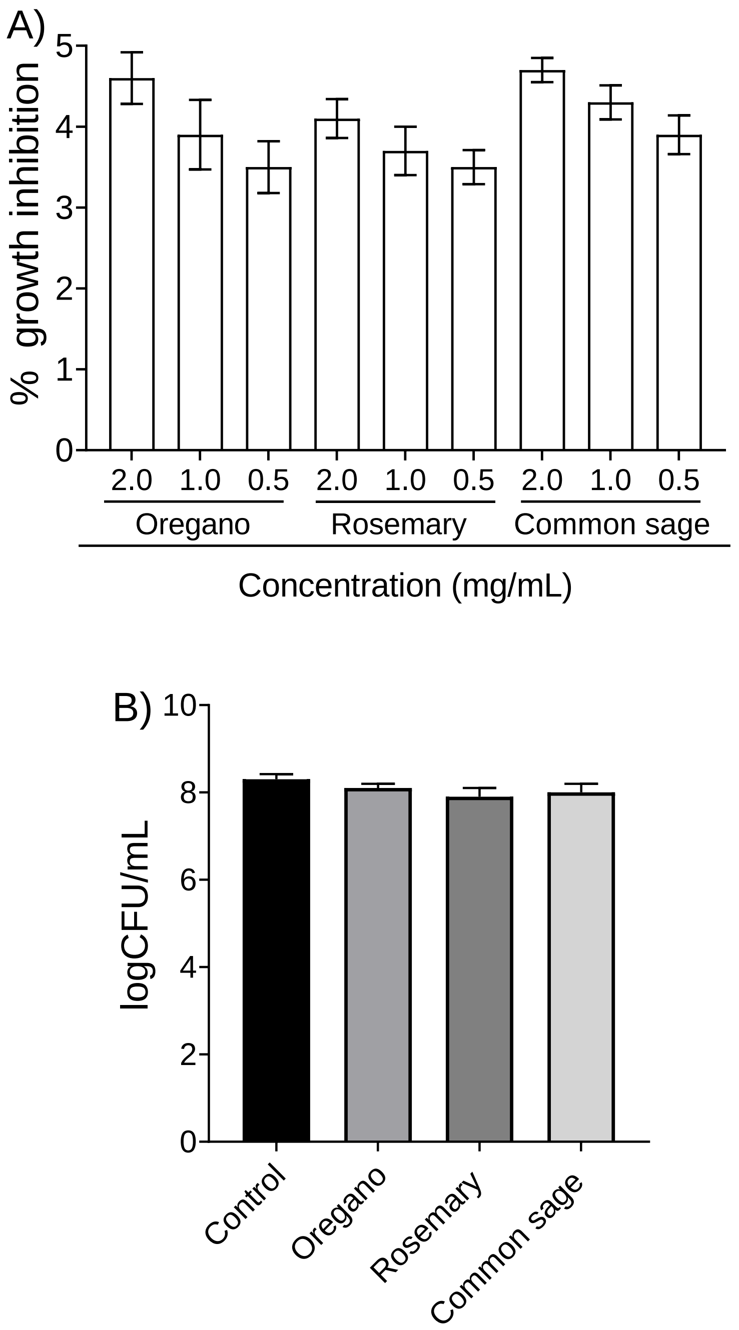

Figure S2: Effect of the ethanolic extracts of oregano, rosemary, and common sage on *E. coli* MG1655 growth. **A)** *E. coli* MG1655 was incubated in the absence and presence of different concentration of the extracts in the wells of a 96 well plate (200 µL per well) for 20 h. **B**) The effect **of** ethanolic of the ethanolic extracts (at 1mg/mL for oregano and rosemary and 2 mg/mL for common sage) on bacterial growth was determined using viable plate counts after 24 h of incubation. Results are presented as mean ± SD, n = 3.

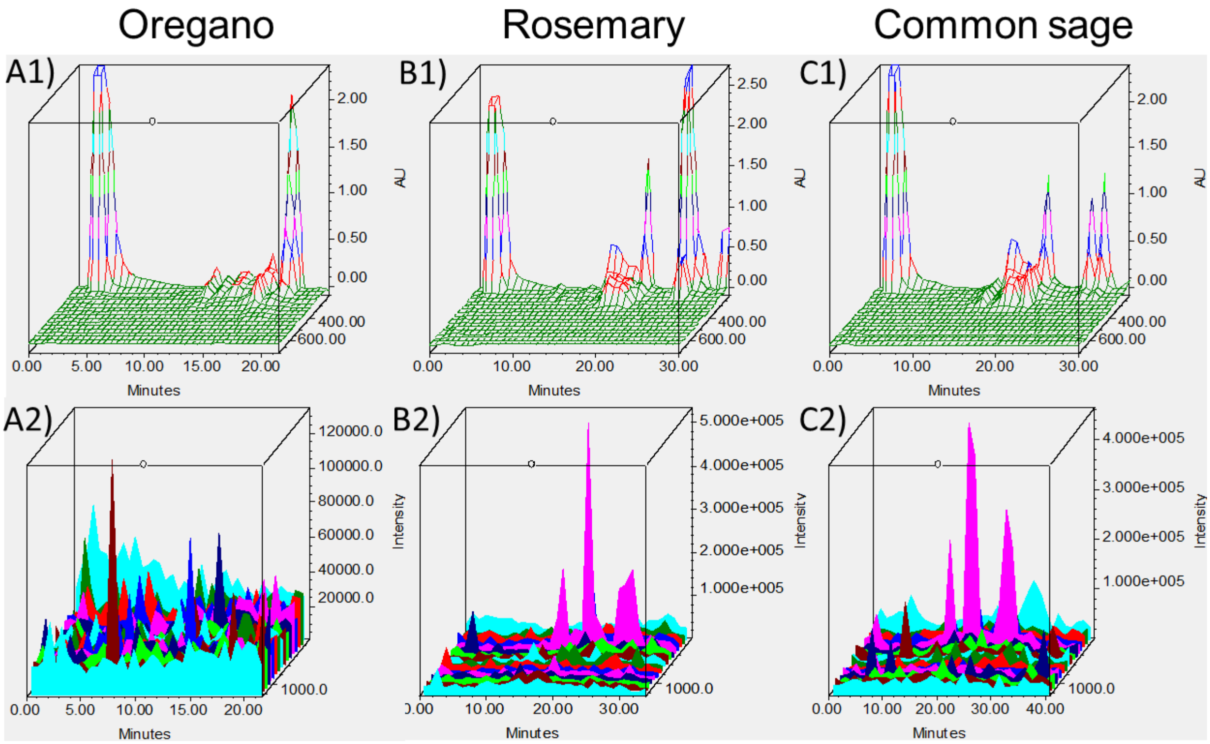

Figure S3: 3D-Chromatograms of extracts obtained using PDA data. Data for oregano (A1, A2), rosemary (B1, B2), and common sage (C1, C2) obtained using PDA data at i) PDA UV-Vis, 210-700 nm (top panels) and, ii) MS full scan (bottom panels).

(A)m/z=271 Apigenin

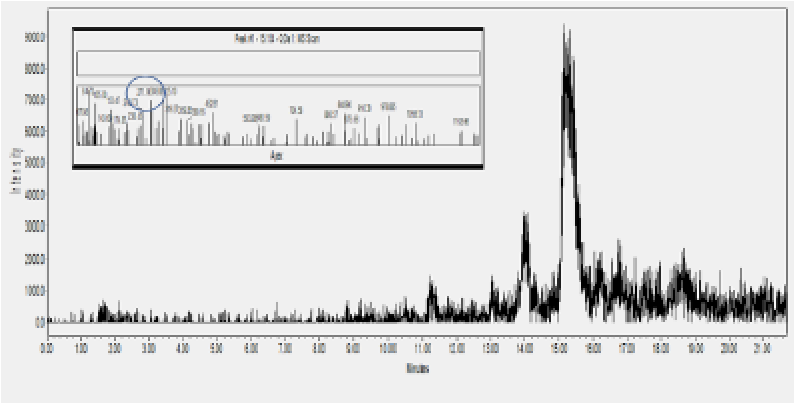

(B)m/z=361 Rosmarinic acid

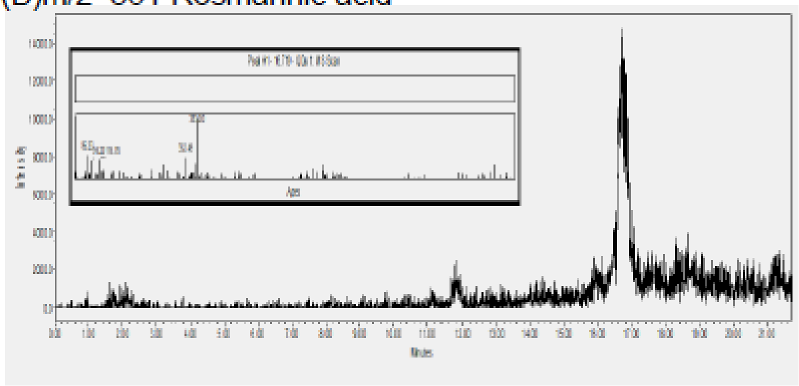

C)m/z=355 Chlorogenic Acid

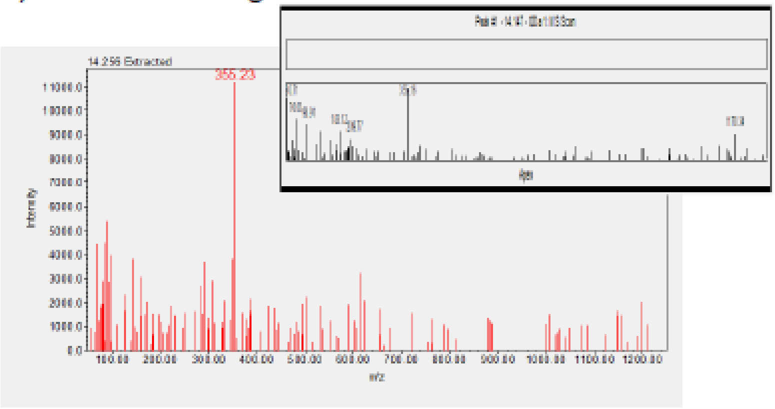

(D) m/z=331 Carnosol

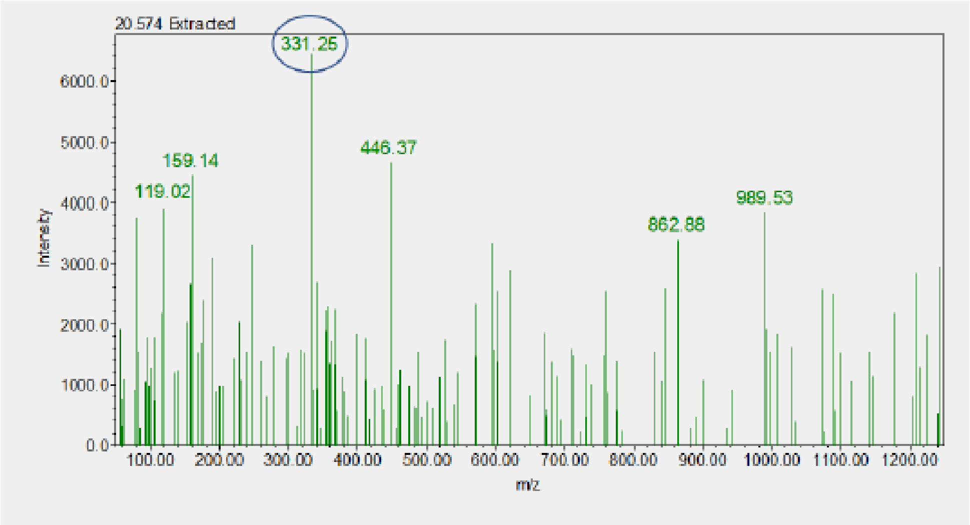

(E) m/z=339 Quercetin

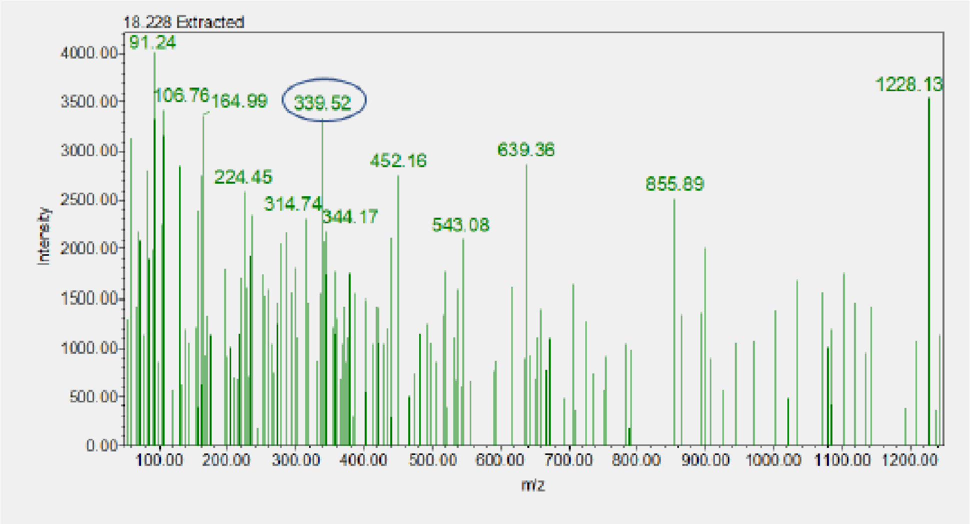

Figure S4: Fragment ions at MS for the ethanolic extracts of oregano

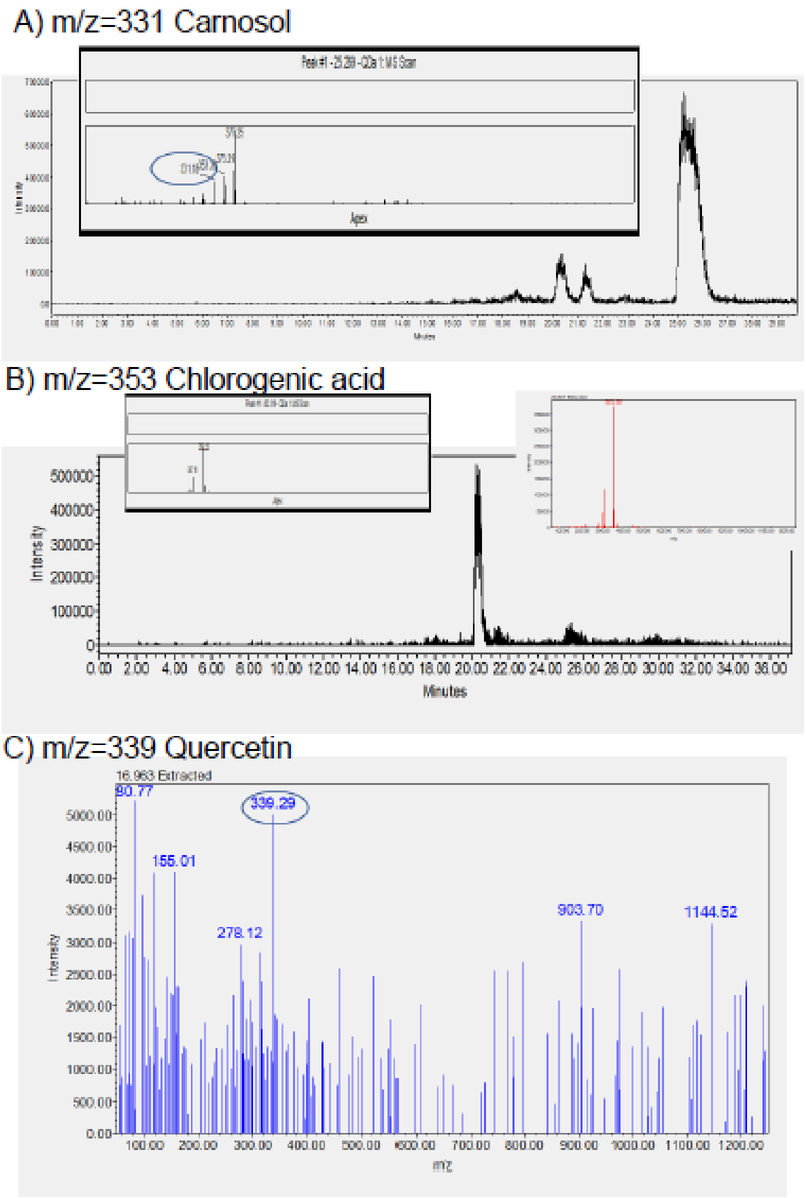

Figure S5: Fragment ions at MS for the ethanolic extracts of rosemary

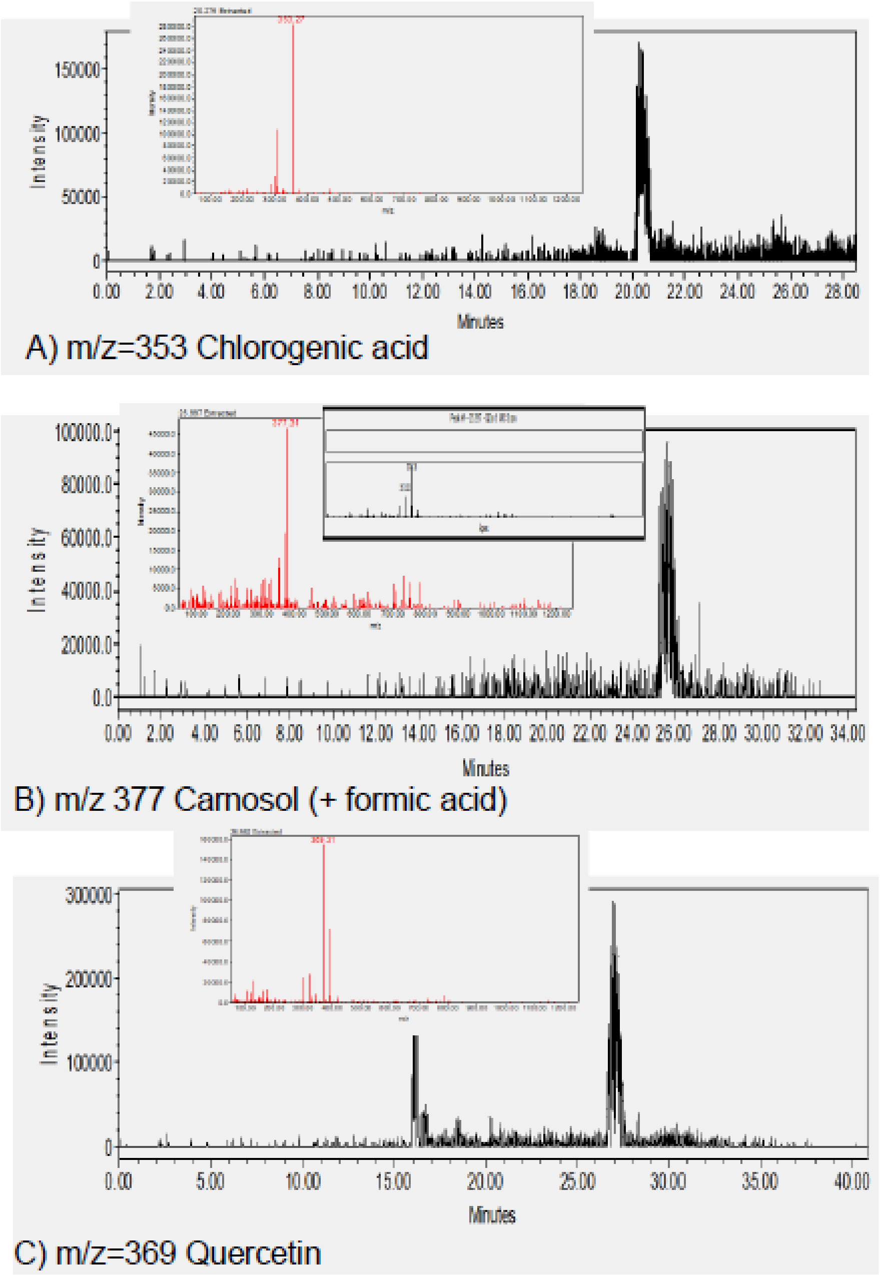

Figure S6: Fragment ions at MS for the ethanolic extracts of common sage
